# Consumption coagulopathy in acute aortic dissection: principles of management

**DOI:** 10.1186/s13019-017-0613-5

**Published:** 2017-06-12

**Authors:** Yuyong Liu, Lu Han, Jiachen Li, Ming Gong, Hongjia Zhang, Xinliang Guan

**Affiliations:** Department of Cardiac Surgery, Beijing Aortic Disease Center, Beijing Anzhen Hospital, Capital Medical University, Beijing Institute of Heart Lung and Blood Vessel Diseases, Beijing Lab for Cardiovascular Precision Medicine, and Beijing Engineering Research Center of Vascular Prostheses, No. 2 Anzhen Street, Beijing, 100029 China

**Keywords:** Acute aortic dissection, Clotting factors, Consumption coagulopathy, Fibrinogen, Platelets

## Abstract

**Background:**

The effect of acute aortic dissection itself on coagulopathy or surgery-related coagulopathy has never been specifically studied. The aim of the present study was to perioperatively describe consumption coagulopathy in patients with acute aortic dissection.

**Methods:**

Sixty-six patients with acute type A aortic dissection were enrolled in this study from January 2015 to September 2016. Thirty-six patients with thoracic aortic aneurysms were used as a control group during the same period. Consumption coagulopathy was evaluated using standard laboratory tests, enzyme-linked immunosorbent assay and thromboelastograghy at five perioperative time-points.

**Results:**

A significant reduction in clotting factors and fibrinogen was observed at the onset of acute aortic dissection. Enzyme-linked immunosorbent assay and thromboelastograghy also revealed a persistent systemic activation of the coagulation system and the consumption of clotting factors. In contrast, although platelet counts were consistently low, we did not find that platelet function was more impaired in the acute aortic dissection group than the control group.

**Conclusions:**

After surgery, clotting factors and fibrinogen were more impaired than platelet function. Thus, we proposed that hemostatic therapy should focus on the rapid and sufficient supplementation of clotting factors and fibrinogen to improve consumption coagulopathy in patients with acute aortic dissection.

## Background

Although substantial advances in surgical techniques such as selective cerebral perfusion and hypothermic circulatory arrest (HCA), have helped to improve early and long-term outcomes, aortic arch surgery in patients with acute Stanford type A aortic dissection remains associated with high morbidity and mortality rates [1, 2]. Postoperative bleeding, allogeneic blood product transfusion and surgical reoperation for bleeding are still some of the most common and feared complications [3, 4]. However, the pathology and mechanisms of acute aortic dissection (AAD) or surgery-induced coagulopathy in aortic arch surgery has not been specifically studied.

Our objective was to provide a detailed description of the state of the perioperative coagulation system and platelets functions in patients with AAD who underwent aortic arch surgery with HCA. This prospective study used standard laboratory tests, enzyme-linked immunosorbent assay (ELISA) and thromboelastography (TEG) to evaluate the basic pathological changes of AAD and the influence of surgery on the coagulation process and platelets functions in patients undergoing emergent aortic arch surgery.

## Methods

### Patient population

From January 2015 to September 2016, a total of 66 patients aged 18 years or older with acute Stanford type A aortic dissection who underwent emergent aortic arch surgery with HCA at our institution were eligible for inclusion. All aortic arch replacements with or without aortic valve operations were eligible. The exclusion criteria included patients with congenital or acquired coagulation disorders, liver disease, previous surgery at the same site, death prior to planned surgery, stroke or myocardial infarction within 2 months before surgery and the use of an oral anticoagulant or antiplatelet treatment within 2 to 5 days before surgery. Thirty-six patients with thoracic aortic aneurysms who underwent surgery during the same period served as the control group. Patients were recruited on a consecutive basis, on the condition that they agreed to provide informed consent.

### Study design

In this single-center prospective study, we perioperatively analyzed the results of standard laboratory tests, ELISA and TEG parameters in 66 patients with AAD and thirty-six patients with thoracic aortic aneurysm at five different time points. All procedures were performed by the same surgery team. The ethics committee at Anzhen Hospital approved the study protocol (Institutional Review Board File 2014019), and consent was obtained from the patients or their relatives. The primary endpoint of this study was to evaluate the state of the coagulation system and platelet activation at hospital admission, during the operation and during the postoperative period in patients with AAD.

### Surgical procedures

Standard anesthetic management was performed with endotracheal intubation. The procedures were performed via a median sternotomy. A right axillary artery was used for arterial cannulation, and the right atrium was cannulated with a single atriocaval cannula. A left ventricular drain was inserted through the right upper pulmonary vein. After systemic heparinization (300 U/kg bodyweight and maintaining an activated clotting time longer than 480 s), CPB was established. During CPB, temperature-adjusted flow rates of 2.5 L/(min · m^2^) were used, and the mean arterial pressure was generally maintained between 50 and 70 mmHg. Our institutional preference was to perform total arch replacement using a tetrafurcate vascular graft combined with implantation of a specific stented graft into the descending aorta. Right axillary arterial cannulation for antegrade cerebral perfusion (5–15 mL/[kg · min]) has been previously performed in our hospital. Our policy was to completely excise the primary tear according to the extent of disruption in each case. The arch was explored under MHCA at a nasopharyngeal temperature between 20 °C and 25 °C. After completing distal anastomosis, CPB was reinstituted, and the patient was gradually rewarmed to a normal temperature after a 5-min period of cold reperfusion for free radical washout. Proximal anastomosis was then performed.

### Blood samples and coagulation assays

Blood samples were obtained from all participants at five different time points: anesthesia induction (T1), lowest nasopharyngeal temperature (T2), protamine reversal (T3), four hours after surgery (T4) and the first postoperative day (T5). The first 5 mL drawn was discarded to eliminate the dilution effect of the saline. The sample was stored in a citrated blood collection tube. Blood was taken from the central venous catheter or peripheral vein and anticoagulated with sodium citrate to evaluate the coagulation system and platelet activation.

White blood cells, hemoglobin, platelet counts and fibrinogen concentration were monitored by standard laboratory tests. Specific assays were performed to assess the activation of the coagulation system and platelets. Plasma was assayed by the monoclonal antibody-mediated sandwich ELISA technique. Activation of the coagulation system was evaluated by assessing thrombin generation through thrombin-antithrombin III complex (TAT) concentration (normal range, 110 to 170 pg/mL), and platelet activation was evaluated by assessing soluble CD40L levels (normal range, 2.03 to 5.92 ng/mL). Blood samples were centrifuged for 15 min at 3,500 rpm at 4 °C and frozen at −80 °C until assayed. All ELISA assays were performed in duplicate, and the mean value was used for analysis.

### TEG analysis

TEG analysis was performed using the TEG 5000 analyzer (Haemoscope, Niles, IL), according to the manufacturer’s instructions. Immediately before analysis a 1-ml sample of citrated blood was added to a vial containing kaolin. After thorough mixing and without delay, 330-ul aliquots were added to two separate cuvettes, each containing sufficient calcium chloride to reverse the effect of the citrate. One of the cuvettes also contained a surface coating of heparinase to deactivate any heparin in the sample. Both cuvettes were loaded onto the analyzer for simultaneous analysis. These constituted two assays of the same sample: one with heparin and one without heparin.

### Statistical analysis

The normality of the data distribution was tested using the Kolmogorov-Smirnov test. Data were presented as the mean ± standard deviation (SD) for continuous data with a normal distribution, median (25th percentile, 75th percentile) for continuous data with a nonnormal distribution, or a number and percentage for categorical values. For comparison, Student’s *t*-test or the Wilcoxon rank-sum test was used for continuous variables, and the chi-square test or Fisher’s exact test was used for categorical variables. The Spearman’s rank correlation test was used to assess relationships between variables. Statistical significance was defined as *P* < 0.05 level, using two-tailed distributions. All statistical analyses were performed using computer software (SPSS 18.0, SPSS, Inc., Chicago, IL).

## Results

### Baseline characteristics

Patient’ characteristics are shown in Table 1. Overall, there were 76 males and 26 females aged 49.5 ± 11.8 years old in this study. Most of the patients with AAD had chest pain (94.8%) as the predominant preoperative symptom. Hypertension was present in 50 out of the 66 patients in the AAD group (*p* < 0.01). The average duration of time from symptom to intervention was 44 h [25% to 75% interquartile range (IQR), 22 to 164 h]. On admission, clotted false lumens were found by enhanced computed tomography in 42 patients. Forty-nine patients had a dissection that extended below the diaphragm and 17 patients had a dissection that terminated above the diaphragm. Left ventricular ejection fraction was lower in AAD patients than patients with thoracic aortic aneurysms (*p* < 0.01). Not surprisingly, the patients with AAD had more severe preoperative clinical conditions than patients with thoracic aortic aneurysms.

**Table 1:** Preoperative clinical characteristics according to study groups

Characteristics	Acute type A aortic dissection (*n* = 66)	Thoracic aortic aneurysm (*n* = 36)	*p*
Age, yr	48.4 ± 10.8	51.4 ± 13.5	0.25
Male	48 (72.7)	28 (77.8)	0.64
Hypertension	50 (75.8)	8 (22.2)	<0.01
Diabetes mellitus	2 (3.0)	7 (19.4)	<0.01
Bicuspid aortic valve	1 (1.5)	3 (8.3)	0.13
Previous cerebral infarction	4 (6.1)	6 (16.7)	0.16
Coronary artery disease	4 (6.1)	2 (5.6)	1.00
Smoking history	27 (40.9)	8 (22.2)	0.81
Marfan syndrome	3 (4.5)	1 (2.8)	1.00
Creatinine, mg/dL	86.8 (64.9, 99.9)	67.8 (60.5, 75.0)	<0.01
Troponin I, ng/mL	0.19 (0.00, 0.06)	0.15 (0.00, 0.01)	0.70
Aortic root size, mm	40.5 ± 7.4	41.7 ± 6.4	0.74
Aortic regurgitation	30 (45.5)	28 (77.8)	<0.01
Ascend aorta size, mm	44.3 ± 6.6	50.6 ± 9.5	0.15
LVEF, %	49.6 ± 6.9	54.0 ± 9.2	<0.01

Values are mean ± SD, n (%), or median (interquartile range)

*BMI* body mass index, *LVEF* left ventricular ejection fraction, *SD* standard deviation

### Perioperative details

The perioperative clinical details are summarized in Table 2. Overall, the types of aortic arch surgery were composite graft or ascending replacement + total arch replacement using a tetrafurcate vascular graft combined with implantation of a specific stented graft into the descending aorta. As expected, patients with AAD required a longer operation time (8.1 ± 1.8 h), CPB time (210 ± 56 min) and aortic cross clamp time (125 ± 47 min) than patients with thoracic aortic aneurysms. The postoperative clinical outcome was also complicated in these patients, whom had a hospital mortality of 10.6%. The causes of death were intraoperative acute heart failure in 2 patients and multi-organ failure in 3 patients. In addition, 1 patient died from sepsis and 1 patient suffered respiratory failure. As expected, patients with AAD had a higher rate of postoperative bleeding and blood product transfusion than patients with thoracic aortic aneurysms.

**Table 2:** Perioperative data according to study groups

Variable	Acute type A aortic dissection (*n* = 66)	Thoracic aortic aneurysm (*n* = 36)	*p*
Ascending + total-arch replacement	38 (57.6)	-	-
Composite graft + total-arch replacement	28 (42.4)	-	-
Ascending replacement	-	10 (27.8)	-
Composite graft	-	26 (72.2)	-
Operation time, hr	8.1 ± 1.8	4.4 ± 0.5	<0.01
CPB time, min	210 ± 56	131 ± 14	<0.01
Aortic cross clamp time, min	125 ± 47	89 ± 15	<0.01
The duration of HCA, min	27.3 ± 1.1	-	-
Lowest nasopharyngeal temperature, °C	23.0 ± 2.0	30.8 ± 0.8	<0.01
Reoperation for bleeding, *n* (%)	6 (9.1)	4 (11.1)	0.74
Intraoperative blood loss, mL	1416 (1000, 1700)	939 (800, 1000)	<0.01
Total postoperative drainage, mL	2900 (1220, 3580)	618 (400, 700)	<0.01
Packed red blood cells, mL	1415 (600, 1870)	330 (0, 600)	<0.01
Fresh frozen plasma, mL	603 (200, 850)	230 (0, 400)	<0.01
Platelet concentrate, mL	300 (0, 600)	200 (0, 300)	0.05
In-hospital mortality	7 (10.6)	3 (8.3)	1.00
Length of in-hospital, day	16.7 (10.5, 19.0)	17.6 (13.0, 19.0)	0.63
Length of ICU, day	6.3 (1.2, 8.5)	3.2 (0.8, 2.0)	0.02
Postoperative dialysis	13 (19.7)	7 (19.4)	1.00
Low cardiac output syndrome	4 (6.1)	4 (11.1)	0.45
Sepsis	12 (18.2)	2 (5.6)	0.13
Paraplegia	3 (4.5)	0	0.55
Respiratory failure	16 (24.2)	3 (8.3)	0.06
Cerebral infarction or bleeding	7 (10.6)	4 (11.1)	1.00

Values are mean ± SD, *n* (%), or median (interquartile range)

*CPB* cardiopulmonary bypass, *ICU* intensive care unit, *HCA* hypothermic circulatory arrest, *SD* standard deviation

### Standard laboratory tests and TEG results

**Fig. 1:**
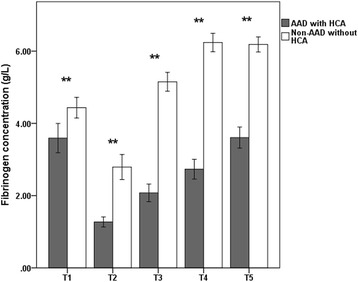
Mean ± SD of the perioperative fibrinogen concentration in patients with AAD and patients with thoracic aortic aneurysms

Changes in standard laboratory tests and TEG results are shown in Table 3. Hemoglobin remained in the low normal range after achieving the lowest nasopharyngeal temperature (T2) in patients with AAD compared to patients with thoracic aortic aneurysms (*p* < 0.01). Moreover, compared to patients with thoracic aortic aneurysms, the platelet count in patients with AAD similarly exhibited the lowest value during surgery and a low level during the postoperative period. In this study, most of the patients exhibited hypofibrinogenemia, and their fibrinogen levels were significantly decreased from the preoperative period to the postoperative period compared with patients with thoracic aortic aneurysms (Fig. 1).

**Table 3:** Standard laboratory tests and TEG results at 5 time points according to study groups

Variable	Acute type A aortic dissection (*n* = 66)	Thoracic aortic aneurysm (*n* = 36)	*p*
Standard laboratory tests			
White blood cells, × 10^3^/μl			
T1**	11.45 ± 3.82	5.32 ± 0.69	<0.01
T2**	4.16 ± 2.51	7.28 ± 6.41	<0.01
T3	10.92 ± 5.59	11.54 ± 4.17	0.56
T4**	10.54 ± 4.27	13.19 ± 2.93	<0.01
T5	14.16 ± 5.56	14.59 ± 4.79	0.69
Hemoglobin, g/dL			
T1	137.03 ± 17.08	136.89 ± 15.35	0.96
T2	78.34 ± 15.44	86.00 ± 22.81	0.08
T3**	89.11 ± 15.01	104.44 ± 13.82	<0.01
T4**	96.17 ± 21.52	110.67 ± 10.87	<0.01
T5**	90.76 ± 16.99	102.67 ± 9.97	<0.01
Platelet counts, × 10^3^/μl			
T1	171.19 ± 54.27	171.22 ± 49.02	1.00
T2**	69.53 ± 33.23	102.56 ± 45.37	<0.01
T3*	96.13 ± 44.77	120.11 ± 47.82	0.01
T4**	91.59 ± 54.05	138.78 ± 41.63	<0.01
T5**	86.65 ± 46.49	120.82 ± 42.49	<0.01
TEG results			
R time			
T1*	6.06 ± 3.44	4.94 ± 1.16	0.02
T2	7.85 ± 1.97	8.25 ± 2.57	0.39
T3	8.03 ± 3.62	7.66 ± 2.84	0.60
T4**	8.10 ± 3.95	6.40 ± 2.33	<0.01
T5*	6.12 ± 2.42	4.97 ± 1.50	0.01
MA			
T1	62.61 ± 7.30	61.96 ± 7.63	0.90
T2	47.14 ± 9.23	46.05 ± 10.92	0.61
T3	51.37 ± 11.81	48.96 ± 9.02	0.29
T4	54.80 ± 10.61	53.64 ± 5.89	0.55
T5	57.44 ± 11.67	60.12 ± 6.49	0.20
α angle			
T1	65.89 ± 9.17	66.54 ± 4.85	0.69
T2	50.69 ± 11.53	51.23 ± 14.67	0.85
T3	54.96 ± 12.60	54.30 ± 12.00	0.80
T4*	54.36 ± 13.62	59.44 ± 8.17	0.02
T5**	62.49 ± 12.58	68.68 ± 3.97	<0.01

Data on the first postoperative day were unavailable for two patients with AAD

T1 = anesthesia induction; T2 = lowest nasopharyngeal temperature; T3 = protamine reversal; T4 = four hours after surgery; T5 = first postoperative day

Values are mean ± SD or median (interquartile range)

*AAD* acute aortic dissection, *MA* maximum amplitude, *TEG* thromboelastography

* *p* < 0.05 ** *p* < 0.01

Preoperative TEG parameters (R time) revealed that the clotting factors were more severely consumed in patients with AAD (*p* = 0.02). Because clotting factors are strongly activated and consumed by surgery and HCA, there was a progressive reduction in clotting factors after surgery (T4 and T5) in patients with AAD (*p* < 0.01 and *p* = 0.01). Compared with patients with thoracic aortic aneurysms, fibrinogen function (α angle) in patients with AAD similarly exhibited the lowest value during surgery and a low level during the postoperative period. However, fibrinogen function recovered more quickly after surgery (T4 and T5) in patients with thoracic aortic aneurysm, whereas its recovery was it is slow in patients with AAD (*p* = 0.02 and *p* < 0.01). Interestingly, although platelet counts were consistently lower in the patients with AAD, there was no difference in platelet function (MA) between the 2 groups (*p* > 0.05).

### ELISA

**Fig. 2:**
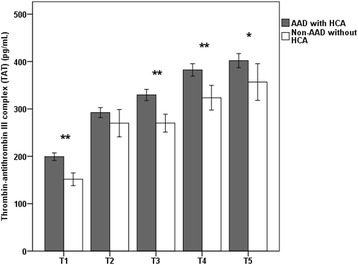
Mean ± SD of the perioperative thrombin-antithrombin III complex (TAT) concentration in patients with AAD and patients with thoracic aortic aneurysms

**Fig. 3:**
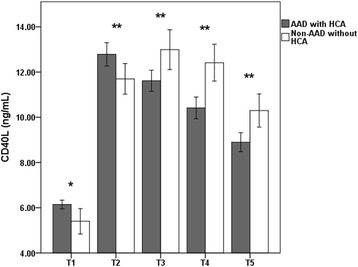
Mean ± SD of the perioperative soluble CD40L concentration in patients with AAD and patients with thoracic aortic aneurysms. The data for the first postoperative day were unavailable for two patients. T1 = anesthesia induction; T2 = lowest nasopharyngeal temperature; T3 = protamine reversal; T4 = four hours after surgery; and T5 = the first postoperative day. Analysis of Student’s *t*-test was *p* < 0.05 for each comparison (**p < 0.01 and *p < 0.05)

The data from patients with AAD showed that TAT levels were markedly elevated before the operation, and they were amplified during CPB, reaching extremely high levels during and after the operation (from 197.18 ± 33.10 to 402.81 ± 62.25 pg/mL) (Fig. 2). Both the AAD and surgery activate coagulation, as reflected by elevated plasma concentrations of TAT. In contrast, soluble CD40L levels dramatically increased to maximum values (12.79 ± 2.08 ng/mL) at the time of the lowest nasopharyngeal temperature (T2) in patients with AAD (*p* < 0.01). However, soluble CD40L levels significantly declined to 9.00 ± 1.63 ng/mL after T2 compared with patients with thoracic aortic aneurysms (*p* < 0.01) (Fig. 3). The soluble CD40L measurements suggested lower platelet activation after T2 in patients with AAD.

## Discussion

Our study specifically described changes in the coagulation system and platelet activation among patients with AAD from symptom onset to the first postoperative day. In this study, we analyzed several molecular biomarkers of the coagulation system and platelet function using ELISA and we analyzed clotting factors, platelet function and fibrinogen function by TEG in patients with AAD who underwent emergent aortic arch surgery. The principal finding of the present study is that AAD itself activates the coagulation system and consumes a large amount of clotting factors and fibrinogen even before surgery. The ELISA results demonstrated that the perioperative procoagulant state is associated with intense thrombin generation (as demonstrated by elevated TAT). In addition, the TEG results also demonstrated consumption coagulopathy (consumption of clotting factors and fibrinogen) in patients with AAD. In contrast, we found that platelet function was not more severely impaired (as demonstrated by soluble CD40L levels and MA) in patients with AAD.

Excessive bleeding is still a common complication that is encountered in aortic arch surgery and often leads to the need for blood transfusions [5, 6]. Thus, surgical treatment of patients with AAD is required to further clarify changes in the coagulation system and platelet functions. The use of CPB in the treatment of AAD has been reported to be responsible for coagulation disorders [7, 8]. Nevertheless, other reports have focused their attention on changes in the perioperative coagulation system during emergent aortic surgery [3, 8], but none of them included a control group to highlight the differences in the coagulation system between patients with AAD and patients requiring complex CPB surgery.

Preoperatively, a low R time suggests clotting factor consumption and, ultimately, coagulopathy in the early phase of AAD. As the final substrate in the coagulation cascade and the ligand of platelet glycoprotein IIb/IIIa receptors, low preoperative fibrinogen levels also demonstrated an initial burst in the consumption of clotting factors. The time from symptom onset to intervention was inversely correlated with the preoperative fibrinogen level (r = 0.63; *p* < 0.01). This is in line with previous findings and may also explain why fibrinogen levels are not always reduced in patients with AAD. Thus, we demonstrated that blood flow contact with the nonendothelialized false lumen results in the release of large amounts of cytokines, which activate the coagulation system and lead to the consumption of many clotting factors before surgery in patients with AAD. This procoagulant state and subsequent consumption coagulopathy at the onset of AAD have been demonstrated, and it eventually results in disseminated intravascular coagulation (DIC)-like coagulopathy in patients with AAD.

As expected, in this study, significantly higher levels of perioperative TAT were observed in AAD patients who underwent aortic arch surgery compared with those with thoracic aortic aneurysms. Therefore, the findings suggest that the procoagulant state and coagulopathy may persist even after the repair of the dissection. This observation suggests a systemic activation of coagulation that potentially lead to impaired hemostasis, which is frequently observed in aortic arch surgery. Through activation of the coagulation cascade, thrombin generation was greatly amplified during CPB despite full heparinization [9, 10]. Because thrombin generation regulates various biochemical and physiological processes involved in coagulation and inflammation, it has a central role in the clotting process [11, 12]. This procoagulant state is a possible underlying mechanism that contributes to microvascular thrombosis, clotting factor consumption and bleeding tendency in patients with AAD [13].

In addition, our TEG results also suggested that the consumption of clotting factors and fibrinogen ultimately lead to coagulopathy and DIC, which was consistent with our previous findings [14]. Thrombus remains in contact with systemic blood flow in AAD through the reverse flow after surgery. This indicates that prolonged contact with a thrombosed area leads to more severe consumption coagulopathy. If bleeding is prolonged, consumption coagulopathy can cause serious complications, such as DIC and multiple organ failure. Thus, we may speculate that massive clotting factor consumption is one of the causes of coagulopathy and bleeding in this type of surgery.

At the same time, our data showed that platelet activation, which was evaluated by measuring the soluble CD40L concentration, was significantly increased in patients with AAD during surgery. The CD40 molecule belongs to the tumor necrosis factor receptor super family that is located at 20q11-13.2. Soluble CD40L is the product of CD40L hydrolysis and is not expressed in non-disease states [15]. CD40L stimulates platelet activation and stabilizes arterial thrombi through a glycoprotein IIb/IIIa ligand-dependent mechanism [16]. At least 95% of circulating soluble CD40L comes from platelets [17]. However, soluble CD40L levels in patients with AAD had a tendency to significantly decline after CPB compared with that in patients with thoracic aortic aneurysms. Moreover, our research confirmed that there was no significant difference in platelet function between the 2 groups during the operation by TEG. This finding revealed that obvious platelet activation and consumption were not observed in patients with AAD. It is possible that the number of platelet glycoprotein IIb/IIIa receptors on the surface of the platelet remained relatively stable after CPB, which is consistent with the findings of previous studies [14, 18]. Indeed, platelet counts and platelet function are separate entities. Additionally, reducing the platelet infusion quantity might also affect clinical prognosis. Thus, we believe that there is no need for excessive platelet transfusion if platelet functions are within normal levels.

Several reports have already reported that that clinical presentations of AAD including anemia, depleting clotting factors and intravascular coagulopathy, result from the consumption of clotting factors in the false lumen [19, 20]. We also confirmed this DIC-like coagulopathy in the acute phase of AAD by ELISA and TEG in this study. Thus, we speculate that the standard guidelines of hemostatic therapy are relatively inadequate for patients with AAD [21]. Based on the different periods of coagulopathy, a suitable medical treatment should involve dealing with the DIC-like coagulopathy. Taking all these factors into consideration, we considered the patients with AAD to be at high risk for perioperative coagulopathy, and their clotting factors and fibrinogen concentrations should be further increased to ameliorate consumption coagulopathy in patients with AAD. Theoretically, thrombin generation should be reduced during the operation, but anticoagulation in patients with an elevated hemorrhagic risk is a difficult task. Therefore, basic scientific principles for this DIC-like state appear to suggest the use of different medications for different phases of AAD.

### Study limitations

A major limitation of our study is that it is still difficult to separate the effects of different pathologies and mechanisms as well as, extended CPB and hypothermia on the coagulation system of the 2 groups. Another important study limitation is that the number of patients and adverse clinical outcomes is small. This small number limited our power of analysis to identify changes in the coagulation system among patients with AAD. In addition, with the experience gained in previous years and with the results of this study, the present authors designed a randomized clinical trial to determine the role of hemostasis in aortic arch surgery.

## Conclusions

The results of this prospective analysis showed that AAD itself activates the coagulation system and consumes a large amount of clotting factors and fibrinogen even before surgery. In our study, these novel data demonstrated that AAD and HCA lead to more severely impaired and consumed clotting factors and fibrinogen than impaired platelet function. Thus, in this consumption coagulopathy setting, we proposed that hemostatic therapy should focus on the rapid and sufficient supplementation of clotting factors and fibrinogen after hypothermic circulatory arrest. According to different periods of coagulopathy, a suitable medical treatment should address the DIC-like coagulopathy.

## Abbreviations

AAD: Acute aortic dissection
CPB: Cardiopulmonary bypass
DIC: Disseminated intravascular coagulation
ELISA: Enzymelinked immunosorbent assay
HCA: Hypothermic circulatory arrest
MA: Maximum amplitude
TAT: Thrombin-antithrombin III complex
TEG: Thromboelastography
